# Engineering of Thermal Stability in a Cold-Active Oligo-1,6-Glucosidase from *Exiguobacterium sibiricum* with Unusual Amino Acid Content

**DOI:** 10.3390/biom11081229

**Published:** 2021-08-17

**Authors:** Yana Y. Berlina, Lada E. Petrovskaya, Elena A. Kryukova, Lyudmila N. Shingarova, Sultan Sh. Gapizov, Mariya V. Kryukova, Elizaveta M. Rivkina, Mikhail P. Kirpichnikov, Dmitry A. Dolgikh

**Affiliations:** 1 Department of Bioengineering, Shemyakin & Ovchinnikov Institute of Bioorganic Chemistry, Russian Academy of Sciences, 117997 Moscow, Russia; yaberlina@gmail.com (Y.Y.B.); kelen.kryukova@yandex.ru (E.A.K.); lshingarova@gmail.com (L.N.S.); gsultan3@gmail.com (S.S.G.); kirpichnikov@inbox.ru (M.P.K.); dolgikh@nmr.ru (D.A.D.); 2 Department of Biology, M.V. Lomonosov Moscow State University, 119234 Moscow, Russia; 3 Kurchatov Complex of NBICS-Technologies, National Research Centre “Kurchatov Institute”, 123182 Moscow, Russia; mar-1-ya@yandex.ru; 4 Laboratory of Soil Cryology, Institute of Physicochemical and Biological Problems in Soil Science, Russian Academy of Sciences, 142290 Pushchino, Russia; elizaveta.rivkina@gmail.com

**Keywords:** oligo-1,6-glucosidase, *Exiguobacterium sibiricum*, proline rule, mutagenesis, cold-active enzymes, permafrost

## Abstract

A gene coding for a novel putative amylase, oligo-1,6-glucosidase from a psychrotrophic bacterium *Exiguobacterium sibiricum* from Siberian permafrost soil was cloned and expressed in *Escherichia coli*. The amino acid sequence of the predicted protein EsOgl and its 3D model displayed several features characteristic for the cold-active enzymes while possessing an unusually high number of proline residues in the loops—a typical feature of thermophilic enzymes. The activity of the purified recombinant protein was tested with *p*-nitrophenyl α-D-glucopyranoside as a substrate. The enzyme displayed a plateau-shaped temperature-activity profile with the optimum at 25 °C and a pronounced activity at low temperatures (50% of maximum activity at 5 °C). To improve the thermal stability at temperatures above 40 °C, we have introduced proline residues into four positions of EsOgl by site-directed mutagenesis according to “the proline rule”. Two of the mutants, S130P and A109P demonstrated a three- and two-fold increased half-life at 45 °C. Moreover, S130P mutation led to a 60% increase in the catalytic rate constant. Combining the mutations resulted in a further increase in stability transforming the temperature-activity profile to a typical mesophilic pattern. In the most thermostable variant A109P/S130P/E176P, the half-life at 45 °C was increased from 11 min (wild-type) to 129 min.

## 1. Introduction

Starch is a storage polysaccharide that is produced in plants as a result of the photosynthetic process. It is used directly as a food component and can be converted into plenty of biotechnologically important products by chemical or enzymatic treatment [1,2]. Specifically, glucose syrup from starch is a raw material for obtaining high-fructose syrup, a popular sweetener in the manufacturing of processed foods and beverages. High product yield at the second stage of the enzymatic starch processing depends on the ability of the used enzymes to efficiently convert oligosaccharides that are present in the reaction mixture into glucose [2]. As a result of inefficient hydrolysis of α-1,6-glycosidic bonds in maltodextrins by the glucoamylase enzyme mainly used at this step, uncleaved by-products are accumulated, thus limiting the final yield.

Oligo-1,6-glucosidase (or isomaltase, EC 3.2.1.10) belongs to the subfamily 31 of the glycosyl hydrolase family 13 (GH13_31) [3] and can hydrolyze α-1,6-glycosidic bonds from the non-reducing end of dextrins and isomaltooligosaccharides. Oligo-1,6-glucosidases (OGLs) are found in different organisms including bacteria, yeasts, filamentous fungi, and animals. All studied OGLs are intracellular enzymes with a molecular weight of 33–71 kDa [3]. Usually, they are active in the temperature range 30–60 °C at slightly acidic or neutral pH (5.0–7.0). The most characterized OGL from mesophilic Bacillus cereus (BceOgl) displays maximum activity at 40 °C [4]. Numerous OGLs from thermophilic microorganisms are described, including those from B. coagulans, B. thermoglucosidasius, and B. flavocaldarius (temperature optima 62, 70–75, and 87.5 °C, respectively) [5,6,7]. However, only several examples of OGLs that are active at low or ambient temperature are reported to date. Such enzymes can be used to achieve efficient starch saccharification at ambient temperature during food and biofuel production [3].

Cold-active enzymes possess high catalytic activity at low temperatures as well as decreased thermal stability [8]. Typically, these molecules demonstrate increased exposure of the hydrophobic residues, reduced size of the hydrophobic core, and weakened intramolecular bonds that confer a higher level of flexibility in comparison with thermostable homologs [9,10,11]. They are usually produced by cold-adapted organisms as a part of their adaptation strategy to survive in cold environments [12,13,14]. Permafrost is one of those ecological niches on Earth that is characterized by permanently low temperature, a limited amount of available nutrients, low water activity, and other extreme conditions. However, it contains diverse microbial communities which have been extensively studied for the last years [15,16,17,18,19]. Particularly, numerous enzymes from permafrost bacteria were produced and characterized that allowed to reveal specific structural and functional features enabling them to catalyze reactions in this environment [20,21,22,23,24]. 

The biotechnological application of the cold-active enzymes offers important advantages, including reduced energy consumption due to their ability to catalyze reactions at ambient temperature and the possibility to work with heat-labile compounds; however, it is limited by the low thermal stability of these enzymes [25,26,27]. Therefore, engineering the cold-active enzymes with a wider temperature range and higher process stability is highly desirable [28,29,30,31]. In this paper, we have obtained and characterized a novel cold-active oligo-1,6-glucosidase EsOgl from a psychrotropic bacterium *Exiguobacterium sibiricum* (Exig_1739 in KEGG database) isolated from Siberian permafrost soil. This microorganism is known for its complex system of adaptation to permafrost conditions [32]. In order to increase the thermal stability of EsOgl, we mutated several residues of EsOgl to prolines and studied the properties of the obtained mutants. We have shown that mutations at certain positions resulted in significant thermal stabilization of EsOgl and a combination of these mutations allowed us to obtain a variant with a 12-fold increased half-life time at 45 °C.

## 2. Materials and Methods

Reagents from Bio-Rad (Hercules, CA, USA), Merck (Darmstadt, Germany), Panreac (Barcelona, Spain), *p*-nitrophenyl α-D-glucopyranoside (pNPG, Sigma St. Louis, MO, USA) and organic solvents (Chimmed, Moscow, Russia) were used in the study. Solutions were prepared using MilliQ water.

### 2.1. Bioinformatic Analysis

Multiple protein alignment was performed with ClustalOmega [33] and visualized with Jalview 2.9.0. Amino acid content was analyzed with ProtParam [34]. A structural model of EsOgl based on homology with *Bacillus cereus* (PDB 1UOK) and *Listeria monocytogenes* (PDB 5DO8) oligo-1,6-glucosidases (66% amino acid sequence identity with EsOgl) was generated using SWISS-MODEL server [35]. 

### 2.2. EsOgl Gene Cloning and Expression

DNA manipulations were performed by standard methods in *Escherichia coli* strain XL-1 Blue using enzymes from Thermo Fisher Scientific, Waltham, MA, USA). Primers were synthesized by Evrogen (Moscow, Russia). 

Gene coding for putative oligo-1,6-glucosidase was amplified by PCR with *Pfu* DNA-polymerase from genomic *E. sibiricum* DNA as a template using primers 1739f and 1739r (Table S1). The PCR product was digested with *Nco*I and *Xho*I and cloned into pET28a vector prepared in the same manner. Insertion in the resulting plasmid pET28EsOgl was confirmed by restriction analysis and sequencing (Evrogen).

*E. coli* BL21(DE3) cells transformed with the recombinant plasmid were grown at 37 °C and 250 rpm in 200 mL of LB medium supplemented with kanamycin (25 μg/mL). After the cell culture optical density reached 0.8 at 560 nm, expression was induced with 0.2 mM IPTG and the culture was incubated at 25 °C and 200 rpm for 4 h. Induced cells were harvested by centrifugation (7000 rpm, 15 min at 4 °C) and resuspended in buffer A (20 mM Tris-HCl, 200 mM NaCl, pH 8.0). The cell suspension was sonicated using a Branson Sonifier 450 on ice for 5 min (15 s on, 1 min off). Cell debris was removed by centrifugation at 17,000 rpm for 15 min. 

### 2.3. Protein Purification

Supernatant after centrifugation was applied to a disposable column filled with Ni-Sepharose Fast Flow resin (GE Healthcare, Chicago, IL, USA) which was equilibrated with buffer A containing 10 mM imidazole. The column was extensively washed with buffer A containing 20 mM imidazole. The target proteins were eluted with buffer B (20 mM Tris-HCl, 200 mM NaCl, 100 mM imidazole, pH 8.0). Combined fractions containing the target protein with a purity of more than 90% were dialyzed against buffer C (20 mM Tris-HCl, 100 mM NaCl) and sterilized. Protein concentration was measured using a Protein Assay Kit (Bio-Rad) with bovine serum albumin as a standard.

### 2.4. Biochemical Characterization of the Enzyme

Oligo-1,6-glucosidase activity measurements were performed using *p*-nitrophenyl α-D-glucopyranoside (*p*NPG) as a substrate. The purified protein (0.5 μg) was added to 0.5 mL of the reaction mixture containing 33 mM potassium phosphate buffer, pH 7.0, 0.36 mM *p*NPG. The mixture was incubated for 15 min at 25 °C, and the reaction was terminated by adding 100 μL of 1 M Na_2_CO_3_. After centrifugation at a table-top centrifuge (13,000 rpm, 5 min), aliquots of the mixture were transferred to the 96-well assay plate (Deltalab, Barcelona, Spain). Absorbance at 405 nm was measured with Multiscan FC reader (Thermo Fisher Scientific). One unit of activity was determined as an amount of enzyme releasing 1 μmol of *p*-nitrophenol per minute. The specific activity was calculated as a number of activity units per 1 mg of protein. All measurements were performed in triplicate. 

The optimal reaction temperature was determined by measuring enzyme activity at different temperatures (5–50 °C). The reaction mixtures without the enzyme and substrate were first equilibrated at the desired temperature for 10 min and then the activity was measured as described above. The thermal stability of the enzyme was determined by measuring residual activity under standard conditions after incubation of the enzyme at different temperatures (from 5 to 50 °C) for 60 min. The half-time of inactivation, *t*_1/2_, was calculated from the time-course study as the first-order kinetics. 

To determine the pH optimum, 50 mM sodium acetate (pH 4.5–5.5), 50 mM potassium phosphate (pH 6.0–8.0), or 50 mM Tris-HCl (pH 8.0–9.5) buffers were used. The effect of salt concentration was measured by adding 0–0.6 M NaCl to the reaction mixture. The effects of various metal ions and other additives on the enzyme activity were evaluated after pre-incubating the enzyme at 5 °C for 30 min in the buffer supplemented with 1 mM solution of each of the additives or in the buffer containing 0.5% and 0.05% (*w*/*v*) of various detergents (SDS, Triton X-100, Tween 20, and CHAPS) at 5 °C and then measuring the enzyme activity according to the standard protocol. The effects of various organic solvents (methanol, ethanol, acetonitrile, DMSO, and DMFA) were estimated by determining residual activity after 30 min incubation at 5 °C in the buffer containing 10 or 30% (*w*/*v*) of each solvent and then measuring the enzyme activity according to the standard protocol. 

Kinetic measurements were performed according to the standard reaction protocol in the presence of 0.4 μg/mL of EsOgl. Kinetic parameters of the reaction were determined from Michaelis-Menten equation using *p*NPG substrate concentrations ranging from 0.5 to 3 mM. The values for *K*_m_ and *V*_max_ were estimated by nonlinear regression using Origin 8 software.

### 2.5. Construction and Characterization of the Mutant Variants

To construct the genes coding for the mutant variants, the Phusion Site-Directed Mutagenesis kit (Thermo Fisher Scientific) was used. PCR with corresponding primers (Table S1) and pET28EsOgl as a template and *Dpn*I digestion of the product were performed according to the manual. The resulting linear product was introduced into *E. coli* cells by electroporation (MicroPulser, Bio-Rad). Plasmid DNA from the obtained clones was isolated and sequenced (Evrogen). Purification of the mutant proteins and their biochemical characterization were conducted as described for the wild-type protein above.

### 2.6. SEC Analysis

Size exclusion chromatography (SEC) of the purified proteins was conducted on a Superdex 75 10/300 GL column (GE Healthcare) at a flow rate of 0.4 mL/min in 100 mM Tris-HCl, pH 8.0, 150 mM NaCl.

### 2.7. Circular Dichroism Spectra

CD spectra were recorded on a Chirascan CD spectrometer (Applied Photophysics, Leatherhead, UK) at 180–320 nm at a constant time of 3 s, scan speed 10 nm/min. Spectra were measured at 20 °C in the protein sample with concentration 0.6 mg/mL in 20 mM K_2_PO_4_, 150 mM KF. The content of the secondary structure elements was calculated with the DichroWeb service [36] using the CDSSTR method and reference data sets 3, 4, 6, 7, SP175, and SMP180. 

## 3. Results

### 3.1. Sequence Analysis and Gene Cloning of EsOgl

**Figure 1:**
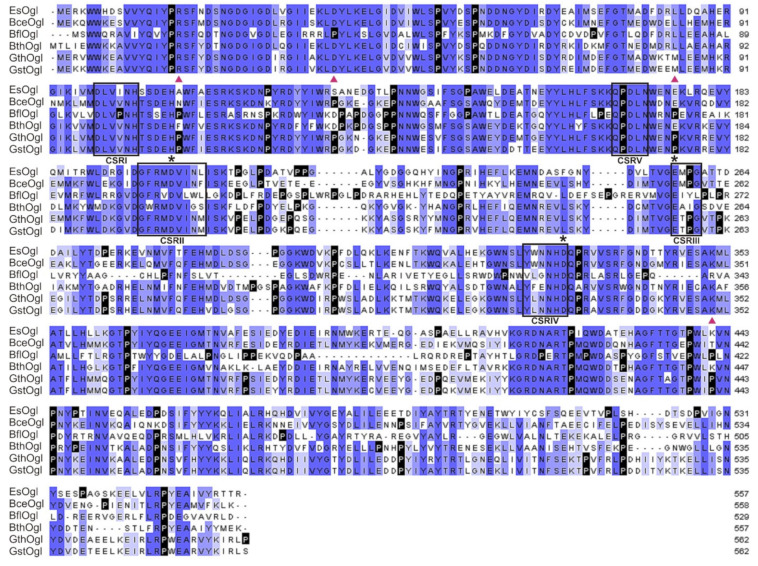
Multiple alignment of EsOgl amino acid sequence with the sequences of homologous oligo-1,6-glucosidases using Clustal Omega. BceOgl, oligo-1,6-glucosidase from *B. cereus*; BflOgl, oligo-1,6-glucosidase from *B. flavocaldarius*; BthOgl, oligo-1,6-glucosidase from *B. thermoamylovorans*; GthOgl, oligo-1,6-glucosidase from *Geobacillus thermoglucosidasius*; GstOgl, oligo-1,6-glucosidase from *G. stearothermophilus*. Proline residues are shown in black. Amino acid residues that have been substituted in the present work are marked with red triangles, and those comprising the catalytic triad with asterisks. Conserved sequence regions (CSRI-V) for the GH13 family and for oligo-α-1,6-glucosidases are boxed.

A gene (Exig_1739 in KEGG database) coding for a putative oligo-1,6-glucosidase (EsOgl) was identified in the genome of *E. sibiricum* [32]. Its nucleotide sequence consists of 1674 bp and encodes a protein of 557 amino acid residues with a predicted molecular weight of ~64.6 kDa. Analysis with SignalP tool revealed the absence of a typical secretion signal sequence indicating the cytoplasmic localization of the predicted enzyme in the host bacterium. The amino acid sequence of EsOgl displayed maximal similarity (61–95%) with enzymes from other *Exiguobacterium* species, and related Gram-positive bacteria including those from genera *Bacillus*, *Laceyella*, *Paenibacillus*, and others. Comparison with the well-studied oligo-1,6-glucosidase from *Bacillus* *cereus* (64% of identical residues) and its functionally characterized homologs allowed identification of the catalytic triad (Glu257, Asp200, and Asp331) and several motifs characteristic for the GH-13 α-amylase family (Figure 1). Specifically, the presence of the fifth conserved sequence region (CSRV) confirmed the annotation of the predicted protein as an oligo-1,6-glucosidase [37].

Analysis of the amino acid content in the protein sequence of EsOgl revealed the signatures of cold-adapted phenotype including increased content of polar residues (Ser, Thr, Gln) and aspartic acid in comparison with oligo-1,6-glucosidases from mesophilic *B. cereus* (BceOgl), and thermophilic *G. thermoglucosidasius* (GthOgl). The content of arginine residues in this protein was lower than in the sequence of GthOgl; however, it exceeded by 1.2% the value of BceOgl. Unexpectedly, the content of proline residues in the sequence of EsOgl was by 1.6% higher than in BceOgl. The number of lysine residues in EsOgl was lower in comparison with both homologs (Table S2).

**Figure 2:**
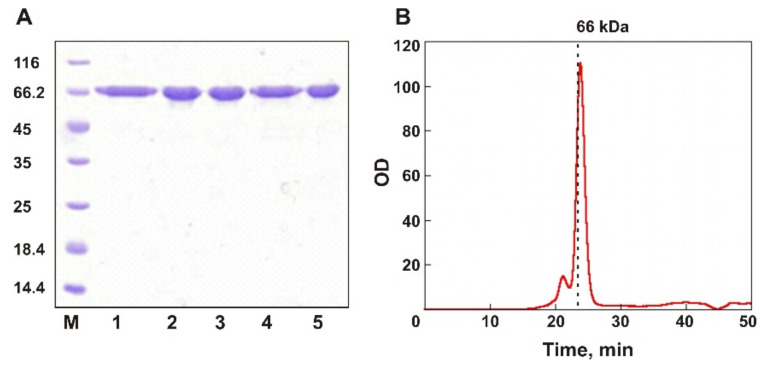
(**A**) SDS-PAGE in 12% gel of the purified EsOgl (lane 1) and the mutants A109P, S130P, E176P, and K441P (lanes 2–5). M, molecular weight markers (kDa, Thermo Scientific). (**B**) SEC analysis of EsOgl on a Superdex 75 column. The dashed line indicates the elution time of bovine serum albumin (24.3 min, molecular weight 66 kDa).

A full-size gene of EsOgl was amplified from the genomic DNA of *E. sibiricum* using gene-specific primers (Table S1) and cloned into the expression vector pET28a in frame with the C-terminal His-tag coding sequence under the control of the T7lac promoter. For the production of the recombinant EsOgl, *E. coli* BL21(DE3) cells were transformed with pET28/EsOgl and cultivated in the presence of 0.2 mM IPTG for 4 h at 25 °C. The recombinant protein with an apparent molecular weight of ~66 kDa was produced in *E. coli* cells in the soluble form and purified using the Ni-affinity chromatography with the yield of 15 mg from 1 l of culture (Figure 2A). Analytical gel filtration of the purified EsOgl on a pre-calibrated Superdex 75 column demonstrated the retention time of the main peak of 23.8 min which corresponds to the monomeric form of the protein (Figure 2B).

### 3.2. Biochemical Characterization of EsOgl

**Figure 3:**
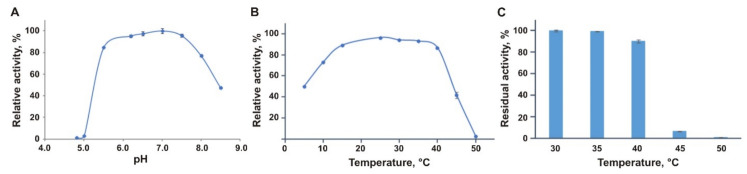
Biochemical characterization of EsOgl. (**A**) Effect of pH on the catalytic activity of EsOgl. Activity in potassium phosphate buffer with pH 7.0 was taken as 100%. (**B**) Effect of temperature on the catalytic activity of EsOgl. Activity at 25 °C was taken as 100%. (**C**) Effect of temperature on the stability of EsOgl. Activity before the incubation at the indicated temperatures was taken as 100%. Mean values of three experiments are presented ± RSD.

The activity of the purified EsOgl was assayed spectrophotometrically with *p*-nitrophenyl-α-D-glucopyranoside (*p*NPG) as a substrate by measuring the increase of the absorbance at 405 nm due to the release of *p*-nitrophenol. EsOgl demonstrated high activity in the pH range 6.0–7.5 in potassium-phosphate buffer (Figure 3A). In Tris buffer, the enzyme was completely inactive at all studied pH values. 

The enzyme showed maximum activity at 25 °C, which is much lower than the temperature optima of the homologous proteins from mesophilic and thermophilic bacteria (41, 62, and 70–75 °C for oligo-1,6-glucosidases from *Bacillus cereus*, *B. coagulans* and *B. thermoglucosidasius*, respectively (Table 1)) [5,6,38]. EsOgl demonstrated a remarkably wide temperature profile retaining over 75% of its maximum activity between 10 and 40 °C, and 50% at 5 °C, unlike, for example, oligo-1,6-glucosidase from *B. cereus* that showed only 25% of the maximum activity at the temperature below 20 °C [38]. At the temperatures above 40 °C, a sharp decrease in enzymatic activity of EsOgl down to 42% at 45 °C was observed, and no detectable activity was detected at 50 °C (Figure 3B). 

The study of EsOgl activity in the presence of different NaCl concentrations (0–600 mM) revealed that it does not affect the activity of the enzyme (data not shown). The influence of other additives on enzyme activity was also investigated (Table 2).

Incubation in the presence of different metal ions at 1 mM concentration and EDTA did not affect the activity of the enzyme, which presumably indicates the absence of bound metal ions in the EsOgl molecule. The addition of PMSF, various detergents, and β-mercaptoethanol also did not produce a profound effect on the activity of EsOgl. The presence of isopropanol, acetonitrile, or acetone in 10% concentration led to a decrease in activity by 33–52% and at 20% concentration by more than 90%. Ethyl and methyl alcohols, as well as DMSO, had a less pronounced inhibitory effect. 

### 3.3. Thermal Stability of EsOgl

The thermal stability of the enzyme was determined by incubation of the protein at different temperatures in the range of 30–50 °C for one hour and measuring its residual activity in the standard assay. It was found that pre-incubation at 30 and 35 °C does not significantly affect the activity of EsOgl. As a result of heating the protein at 40 °C, a slight decrease in the activity was observed (to 90% of the initial value). A sharp drop in activity was observed after incubation at 45 °C (down to 7% of the initial activity), and pre-heating at 50 °C led to almost complete inactivation of the enzyme (Figure 3C).

Studies of the kinetics of EsOgl thermal inactivation at 45 °C (Figure S1A) allowed to determine the inactivation constant, k*_in_*, which was 0.059 ± 0.002 min^−1^, and the half-life time (*t*_1/2_) at this temperature was 11.4 min. Previously reported half-life time for oligo-1,6-glucosidase from *B. cereus* at 45 °C was 6 min [5] which is 47% shorter than that of EsOgl. For the thermophilic oligo-1,6-glucosidases from *B. coagulans* and *B. thermoglucosidasius*, the temperatures at which 50% inactivation of the enzyme occurs after 10 min incubation were 61 °C and 73 °C, respectively [5,6]. Thus, the thermal stability of EsOgl is significantly lower than that of its thermostable homologs, as expected, while it is higher than the thermal stability of oligo-1,6-glucosidase from mesophilic *B. cereus*.

### 3.4. Kinetic Characteristics of the Enzyme

The kinetic constants of the enzyme toward *p*NPG were calculated by nonlinear regression using the Michaelis-Menten equation. Comparison of the obtained results with previously published data revealed that the *K*_m_ value for EsOgl (1.06 ± 0.02 mM) is higher than that for the homologous oligo-1,6-glucosidases (Table 1) [5,6]. This is consistent with the tendency of the cold-active enzymes to have an increased *K*_m_, which is presumably associated with increased mobility of the substrate binding site [8]. The catalytic constant of EsOgl (260 s^−1^) is ~1.8 times lower than the *k*_cat_ of oligo-1,6-glycosidase from *B. cereus* (483 s^−1^). Kinetic parameters for the mentioned enzymes were assayed at different temperatures and, therefore, the obtained results cannot be directly compared. However, considering that the *k*_cat_ value of EsOgl at 25 °C is comparable to the values of the thermostable homologues measured at 50–60 °C (233 and 253 s^−1^ for oligo-1,6-glucosidases *B. thermoglucosidasius* and *B. coagulans* respectively), we can conclude that this enzyme is a highly efficient catalyst at the low-temperature conditions. 

### 3.5. EsOgl Structure Modeling and Site-Directed Mutagenesis

**Figure 4:**
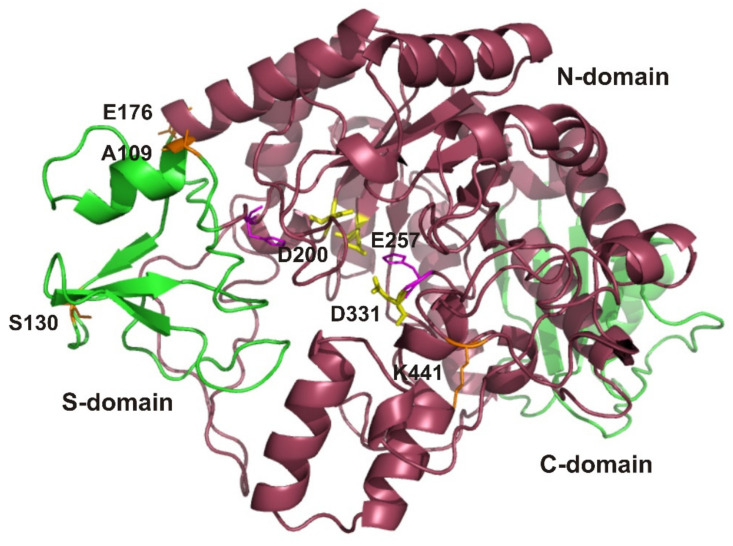
A model of EsOgl 3D structure obtained using the SWISS-MODEL service. The amino acid residues that were chosen for mutagenesis are shown in orange (A109, S130, E176, and K441) and those comprising the catalytic triad are shown in yellow (E257, D200, and D331). Putative N-, S-, and C-domains are colored in purple, light green, and dark green, respectively. The model was visualized with Swiss PDB Viewer.

According to the published data, the presence of proline residues in the loops, at the second positions of the β-turns, and at the N-termini of α-helices increases the rigidity of the protein molecule, thereby improving its thermostability. This so-called proline rule was successfully used to increase the thermal stability of oligo-1,6-glycosidases from *B. cereus* and *B. coagulans* as well as of the enzymes belonging to other families [5,40,41]. In order to increase the thermal stability of EsOgl, we decided to introduce proline residues into the corresponding regions of the enzyme molecule. The 3D structure of the protein was unknown; for that reason, to determine the positions of the potential target residues in respect to the secondary structure elements and to relate them with the homologous thermostable enzymes, we performed homology modeling using the SWISS-MODEL service. The resulting model revealed the typical structure of the enzyme belonging to the α-amylase family consisting of three domains: an N-terminal domain with a TIM barrel topology, a subdomain, and a C-terminal domain (Figure 4) [42].

To identify the target residues for mutagenesis, we have also used the alignment of EsOgl amino acid sequence with the sequences of homologous glucosidases (Figure 1). Consequently, we aimed to introduce proline residues into those positions at which they were present in most of the thermostable glucosidases but were absent in EsOgl. We have also considered the location of the target residues in the secondary structure elements of the protein according to the 3D model (Figure S2). As a result, four residues were chosen for mutagenesis including Ala109, Ser130, Glu176, and Lys441. As evident from Figure 4, two of them (Ser130 and Lys441) are located in the loops, and two others (Ala109 and Glu176) are at the N-termini of the α-helixes. Importantly, the substitutions were planned at the positions remote (at 20–30 Å distance, according to the model) from the residues presumably comprising the catalytic triad in EsOgl to avoid interference with substrate binding and catalysis (Figure 4). 

The mutant genes were obtained by site-directed mutagenesis and expressed in *E. coli* cells according to the protocol developed for the wild-type EsOgl. All isolated mutant proteins demonstrated high purity and electrophoretic mobility, corresponding to the calculated molecular weight of 66 kDa (Figure 2A). The monodispersity of the isolated proteins was assessed by analytical SEC. The elution time of the EsOgl mutant variants from a Superdex 75 column was 23.8–24.0 min, which corresponds to the monomeric form, similar to the wild-type protein (data not shown).

### 3.6. Biochemical Characterization of the Mutant Proteins A109P, S130P, E176P, and K441P

**Figure 5:**
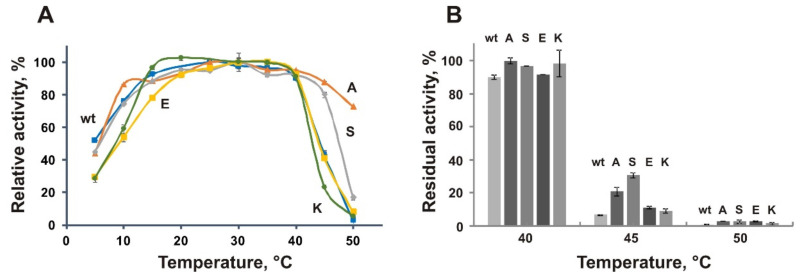
Effect of temperature on the activity (**A**) and stability (**B**) of EsOgl mutant variants. Abbreviations: wt, wild-type EsOgl; A, A109P; S, S130P; E, E176P; K, K441P. Mean values from three experiments are presented ± RSD. (**A**) For the wild-type EsOgl and the mutants, activity at 25 and 30 °C, respectively, was taken as 100%. (**B**) Activity before heating was taken as 100%.

All the obtained mutants demonstrated increased specific activity toward *p*NPG in comparison with the wild-type enzyme (Figure S3). Their level of catalytic activity was almost constant in the temperature range between 20 °C and 40 °C, similar to the wild-type EsOgl (Figure 5A). Remarkably, the A109P and S130P mutants possessed higher activity at 45 °C (more than 80% of the maximum), and A109P retained substantial activity even at 50 °C (52% of the maximum). However, the activity of K441P mutant at 45 °C was 17% lower than that of the wild-type protein. At the same time, the A109P and S130P mutants demonstrated a high level of activity in the low-temperature range. Their activity at 10 °C constituted about 80% of the maximum. As a result, these mutant variants demonstrated a plateau-like temperature dependence that is characteristic for the wild-type EsOgl (Figure 5A).

Similar to the wild-type protein, the mutant variants retained 90–100% of activity after incubation for 1 h at 25–40 °C. However, in contrast to the wild-type EsOgl that maintained only 7% of activity after heating at 45 °C for 1 h, the residual activity of E176P and K441P after such treatment constituted 15% and 14% respectively, and for A109P and S130P it was four- and six-fold higher than that of the wild-type (28% and 42% respectively, Figure 5B). Incubation at this temperature enabled fast and efficient comparison of the mutants and the wild-type EsOgl, therefore studies of the thermal stability were subsequently performed at 45 °C.

Assessment of the kinetics of thermal inactivation of EsOgl mutant variants (Figure S1A) at 45 °C revealed increased half-lives of the mutants A109P (*t*_1/2_ 21 min) and S130P (*t*_1/2_ 29 min) that is 1.8- and 2.6-fold longer than that of the wild-type protein (11 min). The E176P mutant demonstrated only a moderate increase in the half-life at 45% and the half-life of the K441P (12 min) was almost the same as of the wild-type protein (Table 3, find below).

Determination of the catalytic constants of the mutant EsOgl variants was performed similarly to the wild-type enzyme. All mutant variants except K441P demonstrated higher *K*_m_ values in comparison with the wild-type EsOgl. For A109P it was 34% higher, while for S130P and E176P *K*_m_ values increased 1.6- and 1.9-fold, respectively. *K*_m_ value for K441P was similar to that of the wild-type enzyme. *k*_cat_ value of K441P mutant was 33% lower than that of EsOgl. The catalytic constants of S130P and E176P exceeded the value of the wild-type enzyme by 67% and 33% respectively and the A109P mutant demonstrated almost the same *k*_cat_. The catalytic efficiency of S130P was slightly higher in comparison with the wild-type enzyme while the other two variants demonstrated a decrease of the *k*_cat_*/K*_m_ value by 25% (Table 3). 

### 3.7. Effect of the Combined Mutations

Previously, it has been demonstrated that introduction of multiple proline residues stabilized oligo-1,6-glucosidase from *Bacillus cereus* in a cumulative manner [40]. Therefore, we decided to combine all four mutations stepwise and obtained double A109P/S130P (AS), triple A109P/S130P/E176P (ASE), and quadruple A109P/S130P/E176P/K441P (ASEK) mutants. All three variants were produced in *E. coli* cytoplasm in a soluble form and purified by Ni affinity chromatography similarly to the wild-type EsOgl and the single mutants.

**Figure 6:**
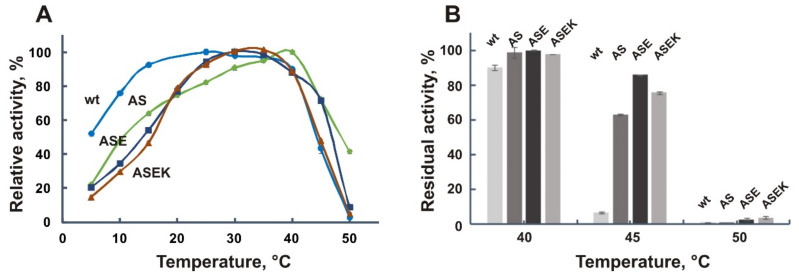
Effect of temperature on the activity (**A**) and stability (**B**) of EsOgl mutant variants with combined mutations. Abbreviations: wt, wild-type EsOgl; AS, A109P/S130P; ASE, A109P/S130P/E176P; ASEK, A109P/S130P/E176P/K441P. Mean values from three experiments are presented ± RSD. (**A**) For the wild-type EsOgl and the mutants AS, ASE, and ASEK, activity at 25, 40, 30, and 30 °C respectively was taken as 100%. (**B**) Activity before heating was taken as 100%.

The temperature optima of activity of the obtained variants shifted to 30–40 °C (Table 3), and their temperature dependences acquired a bell-like shape which is typical for mesophilic and thermophilic enzymes (Figure 6A). The greatest increase of the optimum temperature, up to 40 °C, was observed for the double mutant AS. Accordingly, a decrease in the enzymatic activity of the new mutants at low temperatures was observed. For instance, the relative activity of the ASE and ASEK variants at 10 °C was about 30%, in contrast to the wild-type enzyme, and single mutants A109P and S130P which exhibited more than 70% of the maximum activity at this temperature.

Studies of the thermal stability of the AS, ASE, and ASEK mutants with multiple substitutions revealed its significant increase as a result of the mutations. For instance, after one-hour incubation at 45 °C, the ASE variant retained more than 80% of its activity, while the wild-type enzyme was almost completely inactivated (Figure 6B). Two other mutants demonstrated a slightly lower increase in stability after such heating; the residual activity of the AS mutant constituted 63% and of the ASEK mutant about 76%. 

Finally, to assess the inactivation kinetics, the mutant variants were incubated at 45 °C for two hours in the case of AS mutant and for three hours in the case of ASE and ASEK mutants (Figure S1B). The half-lives of the AS (*t*_1/2_ = 61 min), ASE (*t*_1/2_ = 129 min) and ASEK (*t*_1/2_ = 88 min) mutants were significantly (5.5, 11.7, and 8 times) increased, respectively, in comparison with the wild-type EsOgl (Table 3). Remarkably, the triple mutant ASE was more stable than the ASEK mutant that contained four mutations pointing to the destabilizing effect of the K441P substitution. 

The double mutant AS demonstrated a higher *K*_m_ value in comparison with the wild-type EsOgl (Table 3). For the ASE and ASEK variants, *K*_m_ was lower than that of the wild-type enzyme by 13% and 25% respectively. The catalytic constant *k*_cat_ was lower for all variants with multiple substitutions. As a result, the catalytic efficiency of the mutants slightly decreased in comparison with that of the wild-type EsOgl. For the AS and ASEK mutants, the decrease constituted about 25% and for the ASE it was 13%.

CD measurements of the wt EsOgl and the ASE mutant demonstrated the typical spectra of the α/β hydrolase family members with the large percentage of irregular structure which is characteristic for the cold-adapted proteins. Analysis of the data with DichroWeb service revealed slight differences of the secondary structure of the mutant from that of the wild-type enzyme including the increased content of the alpha-helical structure and decreased amount of the beta-sheets and beta-turns (Table 4).

## 4. Discussion

*Exiguobacterium sibiricum* is a Gram-positive psychrotropic bacterium isolated from three million-year-old Siberian permafrost [43]. According to the results of the transcriptomic and proteomic analysis, this microorganism possesses multiple adaptations which enable its growth and survival in the harsh environmental conditions of permafrost. Of special interest is the ability of *E. sibiricum* to tolerate large thermal fluctuations (from −5 to +40 °C) which are characteristic for this environmental niche [32].

It was shown that *E. sibiricum* prefers sugars and carbohydrates as carbon sources and can degrade starch and glycogen. Consequently, numerous coding sequences for the enzymes which catalyze corresponding biochemical transformations were disclosed in the genome of this bacterium. The expression of several predicted glucosidases was found to be influenced by temperature including Exig_1739 and Exig_2537 genes encoding for the putative alpha-amylases [32]. Expression of the Exig_1739 gene increased at −2.5 °C, while the expression of the Exig_2537 gene was downregulated at this temperature and slightly increased at 10 °C and 40 °C. Analysis of amino acid frequencies in the primary structure of Exig_1739 revealed decreased number of arginine residues and more glycine, lysine, and isoleucine as compared to Exig_2537. Such features are characteristic of cold-active enzymes in comparison with mesophilic and thermophilic homologs [44]. These data suggest that *E. sibiricum* genome contains genes encoding mesophilic and psychrophilic enzymes that can be differently expressed at changing temperature conditions. 

In the current work, we obtained and characterized the recombinant enzyme EsOgl, which is encoded by the Exig_1739 gene and possesses oligo-1,6-glucosidase activity. It has a temperature optimum of activity at 25 °C; however, its activity in the range from 20 °C to 40 °C is also relatively high. As a result, the temperature profile of EsOgl activity is devoid of a pronounced maximum. This property was demonstrated earlier in the studies of several lipolytic enzymes from a psychrotrophic bacterium *Psychrobacter cryohalolentis* K5^T^ [20]. A similar pattern was also reported for the oligo-1,6-glucosidase from *Lactobacillus plantarum* [45]. This feature distinguishes EsOgl from mesophilic and thermophilic homologues, for example, oligo-1,6-glucosidases from *B. cereus*, *B. coagulans* and *B. thermoglucosidasius* that possess bell-shaped temperature profiles of activity with a pronounced maximum [5,6,38]. Consequently, the study of the temperature profile of EsOgl activity confirms that it is a cold-active enzyme in agreement with the prediction made from the analysis of the expression of the corresponding gene and of the amino acid sequence of the protein [32]. In accordance with that, the thermal stability of EsOgl was significantly lower than that of the thermostable homologs. However, its stability upon incubation at 45 °C exceeded that of the oligo-1,6-glucosidase from mesophilic *B. cereus* (BceOgl) (Table 1).

To obtain an explanation for this phenomenon, we have turned our attention to the unusual amino acid content of EsOgl (Table S2). In comparison with BceOgl, the protein sequence of EsOgl contains more arginine and fewer lysine residues (a higher Arg/Lys ration) that is atypical for cold-active enzymes. A low Arg/Lys ratio is observed in the sequences of α-amylase, cellulase, and xylanase from *Pseudoalteromonas haloiplanktis* (AHA), chitobiase from *Arthrobacter* sp. and many other cold-adapted enzymes [8]. However, certain cold-adapted enzymes, including citrate synthase from *Arthrobacter* sp., metalloprotease from *Pseudomonas* sp., and Atlantic cod uracil-DNA glycosylase, demonstrate an increased number of arginine residues in comparison with thermostable homologs. It was proposed that in this case, the arginine sidechains do not participate in stabilizing ionic interactions but rather interact with water molecules, thus increasing molecular flexibility [46].

One of the characteristic features of the cold-active enzymes is the decreased number of proline residues in the loops and their increased amount in the center of α-helices [8]. This is explained by the destabilizing effect of the prolines at latter positions [47,48]. Such tendency was observed in the structures of cold-active DNA ligase, trypsin, alkaline phosphatase, and other enzymes [8]. On the contrary, the presence of the proline residues at the N-terminus of an α-helix promotes its formation [48] and is frequently observed in thermophilic enzymes [7,49]. Consequently, the introduction of additional proline residues at specific positions was proposed as a universal approach to protein stabilization (the proline rule), which has demonstrated its efficiency in numerous experiments [50,51,52]. The total number of prolines in an EsOgl molecule is by eight residues higher than in the homologous enzyme from *B. cereus* and exceeds the number of prolines in the thermostable oligo-1,6-glucosidase from *B. coagulans* by four residues (Table S2). The majority (22 residues) are located in the loops and only three are predicted to reside in the α-helical regions. Several proline residues, whose introduction increased thermal stability of oligo-1,6-glucosidase from *B. cereus* [51], were already present in EsOgl molecule including those at positions corresponding to Lys121, Glu208, Glu216, Glu270, and Glu290 (BceOgl numbering).

To improve the thermal stability of EsOgl for providing its prolonged catalytic activity and shelf-life, we decided to use the proline rule and introduced several amino acid substitutions into this molecule by site-directed mutagenesis. As a result, four variants of EsOgl with point mutations (A109P, S130P, E176P, and K441P) were obtained, as well as three variants containing combinations of these mutations (double A109P/S130P, triple A109P/S130P/E176P, and quadruple A109P/S130P/E176P/K441P mutants). Characterization of the recombinant proteins demonstrated significant differences between their properties and the properties of the wild-type enzyme (Table 3). All mutant variants demonstrated higher specific activity in comparison with the wild-type EsOgl and increased thermal stability. Remarkably, in the most thermostable of the obtained variants (ASE), the half-life at 45 °C increased ~12-fold. In consistence with the known role of the proline residue as a helix initiator [48], this mutant possesses an increased amount of the alpha-helical structure that can be associated with its elevated thermal stability.

Previously, it was shown that temperature adaptation of α-amylases can be provided without changes in the active site of the enzymes [53]. Substitution of the nine residues of *B. cereus* oligo-1,6-glucosidase for the prolines made a significant contribution to the protein stabilization. Moreover, the stepwise addition of the mutations demonstrated an additive effect on thermostability, i.e., provided a consistent increase in thermal stability. The most effective mutations appeared to be located at the second positions of beta-turns and the first positions of alpha-helices. The introduction of the proline residues into the loops led to a less significant stabilizing effect [40].

In the present work, we demonstrated that the introduction of the proline residues at the positions A109 (at the N-terminus of an α-helix) and S130 (in the loop region) resulted in the greatest increase of the thermal stability. Mutation of the homologous residue N109P also resulted in increased thermal stability of oligo-1,6-glucosidase from *B. cereus* [40] and the position homologous to S130 in EsOgl was already occupied with proline in BceOgl (Figure S2). However, the substitution of K441 (also located in the loop) produced almost no impact on the inactivation constant of the enzyme. Combination of the mutations has shown an additive effect in the case of the mutants A109P/S130P (AS) and A109P/S130P/E176P (ASE), while the introduction of the proline residue at the position of K441 in the A109P/S130P/E176P/K441P (ASEK) mutant led to a slight decrease in thermal stability as compared to the triple mutant (ASE). We may speculate that the exposed Lys441 residue side chain is involved in some type of stabilizing interactions with other residue’s side chains and water molecules. Indeed, examination of the obtained EsOgl structure model revealed that the ε-amino group of this residue is situated at a distance of ~2.7 Å from the carboxyl groups of the Asp384 residue that allows the formation of the salt bridge between their sidechains (Figure S4). Certainly, this assumption is tentative and should be verified by the structural investigation of the enzyme.

In conclusion, we have demonstrated that the introduction of the additional proline residues into the EsOgl amino acid sequence not only contributed to a significant increase in thermostability, but also, in certain cases, led to an increased catalytic rate. The *K*_m_ values were concomitantly somewhat increased. However, considering a high substrate load routinely used in industrial applications, an increase in *K*_m_ from 1 mM to 1.5 mM is not relevant. At the same time, the increased catalytic rate can be translated into lower enzyme consumption, shortened reaction time, or a smaller reactor size. Whereas increased thermostability may allow for a wider operational window and increased process stability of the enzyme. These findings confirmed the applicability of the proline rule approach for the cold-active oligo-1,6-glucosidases. However, its efficiency relies on a careful assessment of the individual contribution of each mutation for their subsequent combination in the target protein. In this work, we obtained several mutant variants of EsOgl, ranging from enhanced cold-active enzymes to typical mesophylic ones. These mutant enzymes represent prospective candidates for biotechnological use.

## Figures and Tables

**Table 1:** Comparison of EsOgl biochemical properties with those of the homologous enzymes.

Source of the Enzyme, Reference	Optimum/Assay Temperature, °C	T °C at Which *t*_1/2_ = 10 min	*K*_m_, mM	*k_cat_*_,_ s^−1^	Pro Number
*E. sibiricum* (this work)	20–35 */25	~45 **	1.06	260	28
*B. cereus* [4]	41/35	44	0.80	483	19
*B. coagulans* [5]	62/50	61	0.17	253	24
*B. thermoglucosidasius* [6,39]	70–75/60	71	0.24	233	32

* The temperature range in which the activity was 95–100% of the maximum. ** At 45 °C, *t*_1/2_ = 11.4 min.

**Table 2:** Effect of different additives on the activity of EsOgl. Activity in the absence of additives was taken for 100%.

Additive	Concentration	Relative Activity, %
CaCl_2_	1 mM	99 ± 2
MgCl_2_		103 ± 2
NiCl_2_		105 ± 3
MnCl_2_		98 ± 5
CuCl_2_		94 ± 5
CoCl_2_		94 ± 1
KCl		102 ± 1
β-MЭ		101 ± 2
EDTA		92 ± 1
PMSF		93 ± 4
Triton X-100	0.05%	90 ± 1
Tween-20		89 ± 3
SDS		86 ± 3
Triton X-100	0.5%	92 ± 1
Tween-20		96 ± 1
Ethanol	10%	85 ± 1
Methanol		95 ± 2
iPrOH		67 ± 1
Acetonitrile		48 ± 3
Acetone		56 ± 2
DMSO		79 ± 1
Ethanol	20%	41 ± 1
Methanol		57 ± 1
iPrOH		9 ± 2
Acetonitrile		0.7 ± 0.1
Acetone		9 ± 2
DMSO		35 ± 1

**Table 3:** Biochemical characteristics of the wt EsOgl and the mutant variants.

Enzyme	T_opt_ °C *	Residual Activity after 45 °C, %	*t*_1/2_ at 45 °C, min	*k*_in_, min^−1^	*K*_m_, mM	*k*_cat_, min^−1^ × 10^4^	*k*_cat_/*K*_M_, min^−1^ × mM^−1^ × 10^4^
wt EsOgl	20–35	7	11	0.059 ± 0.002	1.06 ± 0.02	1.5 ± 0.2	1.4
A109P	23–35	28	20	0.033 ± 0.002	1.43 ± 0.09	1.5 ± 0.2	1.0
S130P	20–32	42	29	0.023 ± 0.001	1.66 ± 0.06	2.4 ± 0.3	1.5
E176P	25–37	15	16	0.042 ± 0.002	1.97 ± 0.08	2.0 ± 0.3	1.0
K441P	15–37	14	12	0.055 ± 0.005	0.93 ± 0.08	1.0 ± 0.1	1.1
AS	35–41	63	61	0.0109 ± 0.0002	1.37 ± 0.04	1.3 ± 0.2	1.0
ASE	25–36	86	129	0.0052 ± 0.0001	0.92 ± 0.07	1.2 ± 0.2	1.3
ASEK	27–37	76	88	0.0076 ± 0.0002	0.85 ± 0.02	0.9 ± 0.1	1.0

* The temperature range in which the activity was 95–100% of the maximum.

**Table 4:** Content of the secondary structure elements in the wt EsOgl and ASE mutant variant at 20 °C obtained by CD spectroscopy. Calculations were performed with DichroWeb service [36] using the CDSSTR method and reference data sets 3, 4, 6, 7, SP175, and SMP180.

Protein	α-Helix, %	ß-Sheet, %	β-Turns, %	Unordered, %
wt EsOgl	14.2	34.2	19.6	21.8
ASE	16.4	31.6	17.4	34.4

## Data Availability

The data that support the findings of this study are available from the corresponding author upon reasonable request.

## Supplementary Materials

The following are available online at https://www.mdpi.com/article/10.3390/biom11081229/s1. Table S1: nucleotide sequences of the primers used for construction of the mutant genes, Table S2: the content of selected amino acid residues in the protein sequences of oligo-1,6-glucosidases from *E. sibiricum* (EsOgl), *B. cereus* (BceOgl), and *G. thermoglucosidasius* (GthOgl), Figure S1: kinetics of inactivation of the wild-type EsOgl and its mutant variants at 45 °C, Figure S2: structural alignment of EsOgl model with oligo-1,6-glucosidases from *B. cereus* (PDB 1UOK) and *Listeria monocytogenes* (PDB 5DO8). Figure S3: enzymatic activities of the wild-type EsOgl and the mutant variants. Figure S4: Localization of the sidechains of Asp384 and Lys441 residues in EsOgl 3D structure.
