# Supplementary material for: Engineering of Thermal Stability in a Cold-Active Oligo-1,6-Glucosidase from Exiguobacterium sibiricum with Unusual Amino Acid Content
Source: Biomolecules. 2021 Aug 17;11(8):1229. doi: 10.3390/biom11081229 (PMC8392543; doi:10.3390/biom11081229)
Supplement: Supplementary file 1 [file biomolecules-11-01229-s001.zip › biomolecules-1303225-supplementary.pdf]

Supplementary Material to the manuscript

"Engineering of Thermal Stability in a Cold-Active Oli-go-1,6-Glucosidase from Exiguobacterium sibiricum with Un-usual Amino Acid Content" by Berlina et al.

Table S1. Nucleotide sequences of the primers used in the study for gene construction and mutagenesis. Recognition sequences of restrictases used for gene cloning are underlined.

Primer name	Sequence, 5′-3′
1739f	ATCATA<u>CCATGG</u>AACGTAAATGGTGGCAC
1739r	ACAT<u>CTCGAG</u>CCGCGTCGTCCGGTAGAC
A109Pf	ACCATTCGTCGGACGAGCACCCATGGTTTGCAGAG
A109Pr	GTGCTCGTCCGACGAATGGTTGATGACCAA
S130Pf	GGACTATTACATTTGGAGACCAGCCAATGAAGACGG
S130Pr	TCTCCAAATGTAATAGTCCCGGTATGGATT
E176Pf	CTGATCTGAACTGGGAAAACCCAAAACTGCGTCAAG
E176Pr	GTTTTCCCAGTTCAGATCAGGTTGCTTTTT
K441Pf	CGACGGGTACACCCTGGTTACCAGTTAATCCGAAC
K441Pr	TAACCAGGGTGTACCCGTCGTAAATCCGGC

Table S2. The content of selected amino acid residues in the protein sequences of oligo-1,6-glucosidases from *E. sibiricum* (EsOgl), *B. cereus* (BceOgl), and *G. thermoglucosidasius* (GthOgl). Values were obtained using the ProtParam program.

Residue	Content, %		
	EsOgl	BceOgl	GthOgl
Arg	4.8	3.6	5.9
Asn	5.4	6.6	5.0
Asp	8.4	6.8	8.0
Gln	2.9	2.7	2.5
Glu	8.6	11.1	8.9
Gly	6.6	6.5	6.6
Lys	4.5	7.3	7.5
Pro	5.0	3.4	5.7
Ser	5.4	5.0	5.0
Thr	5.7	4.1	4.1

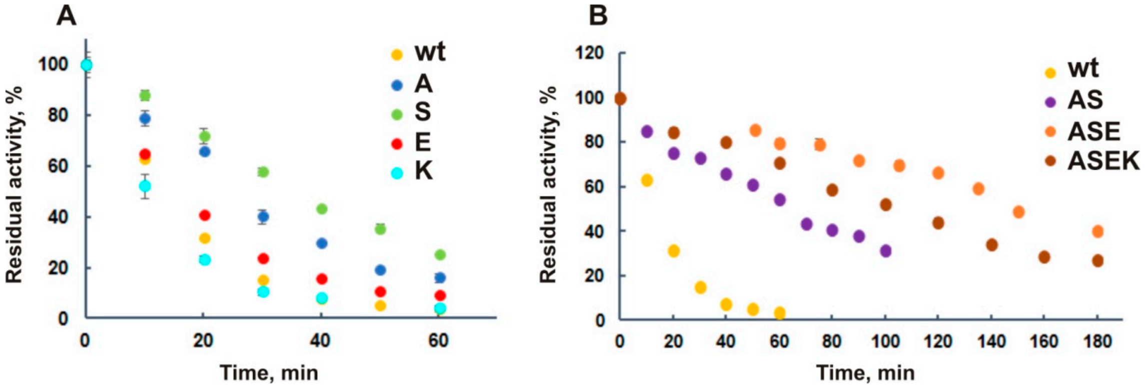

Figure S1. Kinetics of inactivation of the wild-type EsOgl and its mutant variants with single (A) and multiple (B) substitutions at 45 °C. Abbreviations: wt, wild-type EsOgl; A, A109P; S, S130P; E, E176P; K, K441P; AS, A109P/S130P; ASE, A109P/S130P/E176P; ASEK, A109P/S130P/E176P/K441P. Activity before incubation was taken as 100%. Mean values of three experiments are presented ± RSD.

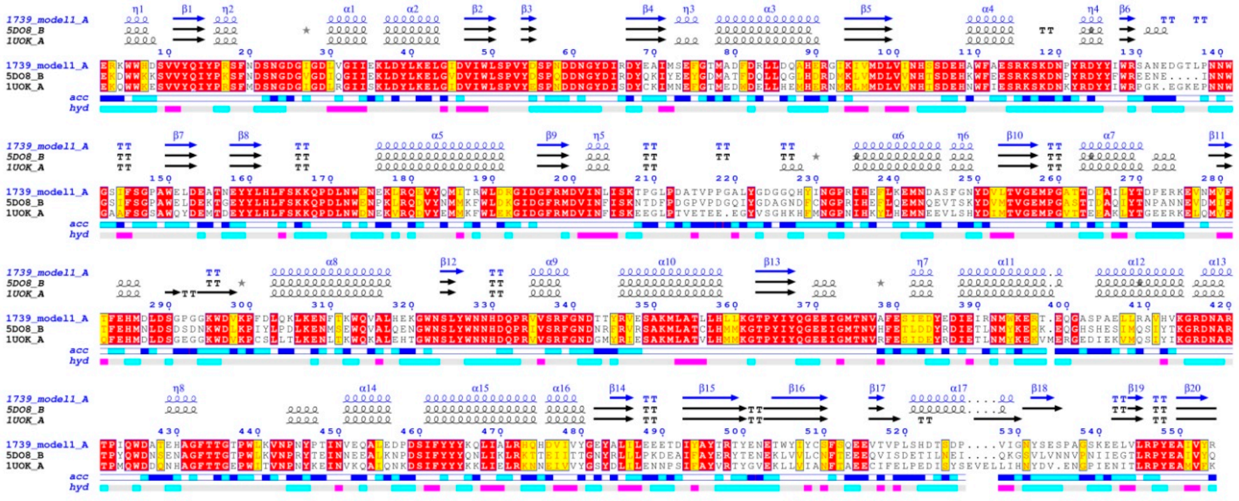

Figure S2. Structural alignment of EsOgl model with oligo-1,6-glucosidases from *Bacillus cereus* (PDB 1UOK) and *Listeria monocytogenes* (PDB 5DO8). Secondary structure elements were identified using ENDscript. Helices are shown by squiggles, strands are indicated by arrows, turns are indicated by TT letters, and 3_{10}-helices are indicated by η letters.

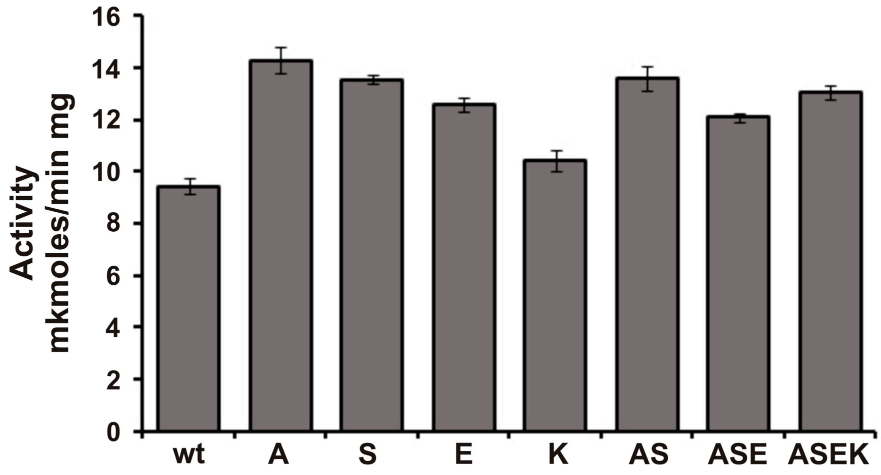

Figure S3. Enzymatic activities of the wild type EsOgl and the mutant variants. The reaction mixture containing 0.36 mM *p*NPG and 1 mkg of indicated protein in 33 mM potassium phosphate buffer, pH 7.0 was incubated for 15 min at 25ºC. One unit of activity was determined as an amount of enzyme releasing 1 µmol of *p*-nitrophenol per minute. The specific activity was calculated as a number of activity units per 1 mg of protein.

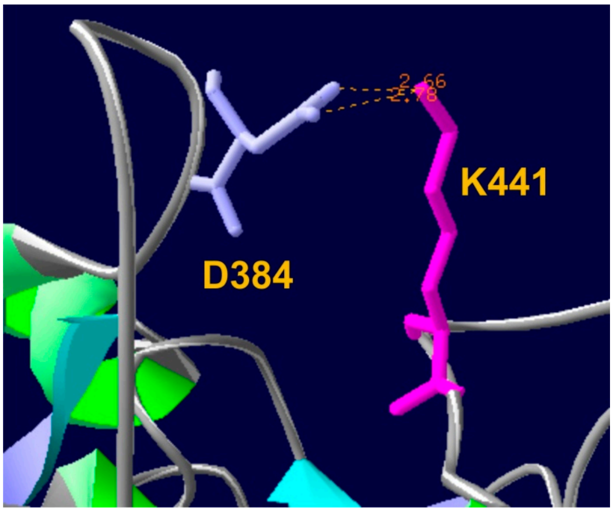

Figure S4. Localization of the sidechains of the Asp384 and Lys441 residues in EsOgl 3D structure. The model of EsOgl 3D structure was obtained using the SWISS-MODEL service and visualized by Swiss PDB Viewer.
